# Selective Serotonin Reuptake Inhibitors and Clozapine: Clinically Relevant Interactions and Considerations

**DOI:** 10.3390/neurolint13030044

**Published:** 2021-09-01

**Authors:** Amber N. Edinoff, Juliana M. Fort, Joshua J. Woo, Christopher D. Causey, Caroline R. Burroughs, Elyse M. Cornett, Adam M. Kaye, Alan D. Kaye

**Affiliations:** 1 Department of Psychiatry and Behavioral Medicine, Louisiana State University Health Science Center Shreveport, 1501 Kings Hwy, Shreveport, LA 71103, USA; juliana.fort@lsuhs.edu; 2 School of Medicine, Louisiana State University Health Shreveport, Shreveport, LA 71103, USA; jwoo@lsuhsc.edu (J.J.W.); ccaus1@lsuhsc.edu (C.D.C.); cburro@lsuhsc.edu (C.R.B.); 3 Department of Anesthesiology, Louisiana State University Health Science Center Shreveport, Shreveport, LA 71103, USA; ecorne@lsuhsc.edu (E.M.C.); alan.kaye@lsuhs.edu (A.D.K.); 4 Department of Pharmacy Practice, Thomas J. Long School of Pharmacy and Health Sciences, University of the Pacific, Stockton, CA 95211, USA; akaye@pacific.edu

**Keywords:** treatment-resistant schizophrenia, SSRIs, augmentation, clozapine, schizophrenia

## Abstract

The monoamine hypothesis of depression attributes the symptoms of major depressive disorders to imbalances of serotonin, noradrenaline, and dopamine in the limbic areas of the brain. The preferential targeting of serotonin receptor (SERT) by selective serotonin reuptake inhibitors (SSRIs) has offered an opportunity to reduce the range of these side effects and improve patient adherence to pharmacotherapy. Clozapine remains an effective drug against treatment-resistant schizophrenia, defined as failing treatment with at least two different antipsychotic medications. Patients with schizophrenia who display a constellation of negative symptoms respond poorly to antipsychotic monotherapy. Negative symptoms include the diminution of motivation, interest, or expression. Conversely to the depressive symptomology of interest presently, supplementation of antipsychotics with SSRIs in schizophrenic patients with negative symptoms lead to synergistic improvements in the function of these patients. Fluvoxamine is one of the most potent inhibitors of CYP1A2 and can lead to an increase in clozapine levels. Similar increases in serum clozapine were detected in two patients taking sertraline. However, studies have been contradictory as well, showing no such increases, which are worrying. Clinicians should be aware that clozapine levels should be monitored with any coadministration with SSRIs.

## 1. Introduction

The monoamine neurotransmitters serotonin (5-hydroxytryptamine, 5-HT), dopamine, and norepinephrine are each generated exclusively by their respective populations of neurons. The homeostasis of the monoamine neurotransmitters is maintained by a family of ATPase active transporters that symport sodium and chloride ions. The monoamine transporters serotonin transporter (SERT), norepinephrine transporter (NET), and dopamine transport (DAT) regulate the concentrations of serotonin, norepinephrine, and dopamine, respectively. NET is located in the plasma membrane of noradrenergic neurons, and its function is to take up released norepinephrine, which was released from the synapse [1]. DAT is responsible for the clearance of dopamine from the extraneuronal space after it is released from vesicles [2].

These transporters are frequent targets of psychoactive medications and drugs of abuse. The relative affinities of these drugs for specific populations of monoamine transporters determine their therapeutic utility, liabilities for off-target effects, and potential for abuse [3]. Pharmacologic manipulation is usually achieved by competitively or allosterically inhibiting the conformational changes of the transporter, preventing the intracellular uptake of neurotransmitter, and causing it to remain available in the synapse at higher concentrations.

The monoamine hypothesis of depression attributes the symptoms of major depressive disorders to imbalances of serotonin, noradrenaline, and dopamine in the limbic areas of the brain. Tricyclic antidepressants showed efficacy in treating depression through their actions on SERT, NET, and DAT. However, this broad range of activity at these receptors gave rise to side effects, including dry mouth, hypotension, and urinary retention. The preferential targeting of SERT by SSRIs offered an opportunity to reduce the range of these side effects and improve patient adherence to pharmacotherapy. Side effects of SSRIs may arise either directly from the inhibition of SERT or from downstream effects of increased synaptic serotonin upon other producers of neurotransmitters. In an example of clinical relevance, it is hypothesized that increased levels of synaptic serotonin may inhibit dopamine-producing neurons, thereby leading to the debilitating movement disorders of extrapyramidal symptoms. Thus, while SSRIs may target SERT almost exclusively, the effects of inhibition are complex and may impinge upon distinct systems of neurotransmission [4]. 

While SSRIs are broadly effective in the treatment of depression, it remains necessary to augment their effects in patients whose symptoms are significantly resistant to SSRIs alone. Several such SSRI-augmentation strategies are available to clinicians, such as lithium and triiodothyronine, both of which show only modest augmentation. When such second-line augmentations prove ineffective, augmentation with atypical antipsychotics may be considered. When considering the potentially serious off-target effects of atypical antipsychotics, such as clozapine, it is important to carefully weigh the risks and benefits of such therapy, especially when the residual disease process includes a durable propensity for suicidal attempts as a result of their illness [5].

As a result of the need for treatment options in these patients, the FDA has approved clozapine for the treatment of schizophrenia that has proven resistant to multiple other treatments and for the prevention of suicide in patients with psychotic disorders. Despite some demonstrated efficacy in the treatment of psychotic and bipolar depression, serious off-target effects, which include leukopenia and granulocytopenia, should be strictly monitored to prevent progression to granulocytopenia. Substantial risks of developing myocarditis, and other adverse cardiovascular and respiratory events, are serious physiological considerations to be weighed against treatment. Since many patients with schizophrenia require more than one medication to control their psychiatric symptoms, it is important to look at medication interactions. Clozapine and SSRIs can have concerning interactions, so this manuscript aims to create a summary for the clinician regarding interactions between these two medications.

## 2. SSRI History, Uses, and Mechanism of Action

### 2.1. History

First proposed by Schildkraut in 1965 [6], the catecholamine hypothesis suggested that behavioral states depended on the amount of catecholamines centrally; depression, therefore, was associated with a deficit of catecholamines, particularly norepinephrine, at certain brain receptors. Monoamine oxidase inhibitors like iproniazid prevented degradation of brain norepinephrine and other amines, including serotonin and dopamine [7,8]. Tricyclic antidepressants (TCAs) such as imipramine inhibited the cellular reuptake of norepinephrine [9]. Feighner’s review of the literature, however, noted that TCAs interact with multiple receptor sites and can result in atropine-like side effects, making TCAs less viable for long-term treatment [10]. MAOIs, too, force patients to adopt a strict diet to prevent tyramine interactions, as these could lead to severe hypertension [10,11,12]. 

SSRIs were introduced as comparable antidepressant drugs to TCAs and MAOIs but with a more tolerable side effect profile. Brambilla et al. conducted a meta-analysis to evaluate the side effect profiles of SSRIs, particularly fluoxetine, against those of TCAs and MAOIs and found that a significantly lower percentage of patients treated with fluoxetine experienced any side effects when compared to those treated with TCAs [13]. Anderson et al. showed that patients treated with SSRIs were less likely to discontinue treatment than those treated with TCAs [14].

### 2.2. Uses

SSRIs are chiefly used to treat major depressive disorder (MDD), but other indications include panic disorder, generalized anxiety disorder (GAD), obsessive-compulsive disorder (OCD), post-traumatic stress disorder (PTSD), social anxiety disorder, and bulimia nervosa [15].

An eight-week open study by Compton et al. concluded that sertraline, a commonly-prescribed SSRI for the treatment of social anxiety disorder, showed a significant reduction in subject-reported symptoms of depression and subjective levels of distress over the course of the trial [16]. Salaminios et al. suggested a randomized, double-blind, placebo-controlled clinical trial protocol for adults aged 18 to 74 presenting to primary care with depression to investigate the severity and duration of depressive symptoms associated with a response to sertraline [17]. The results of this 12-week trial, as reported by Lewis et al. showed a weak association between reduced depressive symptoms at 12 weeks of sertraline treatment compared with placebo [18]. Secondary analyses of these data showed a reduction of generalized anxiety symptoms at 6 and 12 weeks compared with placebo, and self-rated mental health improvement [18]. SSRIs are often effective for both MDD and GAD, as they tend to be comorbid with each other [15].

Fluvoxamine is commonly used to treat severe OCD in adults [19] and was the first to be registered to treat OCD in children [20]. Clomipramine, a tricyclic antidepressant, received FDA approval for the treatment of OCD [21] but exhibited greater total side effects than fluvoxamine [22]. The most effective treatment strategy is a combination of an SSRI with cognitive-behavioral therapy (CBT) [23]; in one study, therapy with paroxetine alone showed an average relapse time of approximately 63 days [24].

Although patients treated for panic disorder did not show SSRIs to be any more effective than benzodiazepines in a 10-year longitudinal study [25], SSRIs are still considered first-line therapy for panic disorder. Citalopram, which received FDA approval in August 1998 [26], was shown to be effective for panic symptoms in an open trial [27]. Similar efficacy against panic disorder was also shown for the enantiomer escitalopram [28], with a similar side effect profile [29]. A 2005 Dutch study of the cost-effectiveness of panic disorder showed that SSRI therapy in combination with CBT is more cost-effective than either therapy alone [30].

Literature supports the treatment of PTSD with SSRIs. Higher doses of escitalopram (40 mg/d) were shown to reduce major depressive symptoms in patients who did not respond to doses up to 20 mg/d in an open-label study [31]. A double-blind, placebo-controlled trial indicated that fluoxetine is also well-tolerated in treating PTSD, with few significant side effects [32].

Fluoxetine received FDA approval for the treatment of bulimia [21]. While some placebo-controlled studies have demonstrated a reduction in behavioral symptoms consistent with bulimia, such as self-induced vomiting and binge-eating [33,34], another placebo-controlled study was limited by a high patient dropout rate [35]. Other studies evaluating fluvoxamine and sertraline in treating bulimia have been performed but are limited by small sample sizes [36,37].

### 2.3. Mechanism of Action

Although the complete mechanism by which SSRIs function is not fully understood, the literature generally agrees upon the initial step, suppression of the 5-HT firing neurons leading to greater availability of synaptic 5-HT within the raphe nuclei [38,39]. Sustained SSRI treatment eventually desensitizes the 5-HT_1A_ autoreceptors (and 5-HT_1B_ autoreceptors after two to three weeks of therapy), which results in more 5-HT being released per action potential [38,39]. Sprouse et al. found that fluoxetine inhibited hippocampal cell firing via 5-HT_1A_ receptor activation, despite a decrease in dorsal raphe cell firing [39].

### 2.4. SSRIs Side Effects and Safety Concerns

Selective serotonin reuptake inhibitors were developed by Eli Lilly as a means of achieving the antidepressant effects of serotonin reuptake inhibition alone, thereby limiting the experience of side effects related to the nonselective inhibition of norepinephrine and dopamine receptors. The improved selectivity decreased the burdens of dry mouth, hypotension, and urinary retention and demonstrated improved clinical utility and patient adherence [4]. Nonetheless, SSRIs themselves retain a range of side effects that have grown in clinical importance as polypharmacy threatens to increase levels of intrasynaptic serotonin above intended limits or to create unintended interactions with other neurotransmitters. 

### 2.5. Serotonin Syndrome

Serotonin syndrome, which results from toxic levels of synaptic serotonin, is becoming an increasingly common clinical problem. The current gold standard for the diagnosis of serotonin syndrome is the Hunter serotonin toxicity criteria to achieve a sensitivity of 84% and a specificity of 97% [40]. These criteria are, in the presence of a serotonergic agent:

1. Spontaneous clonus
2. Inducible clonus and agitation or diaphoresis
3. Ocular clonus and agitation or diaphoresis
4. Tremor and hyperreflexia
5. Hypertonia
6. Temperature > 38 °C and ocular clonus or inducible clonus [41]

The most common cause of serotonin syndrome is the administration of multiple serotonergic drugs that act to increase serotonin synthesis, serotonin receptor agonists, and/or serotonin reuptake inhibitors. Because serotonergic drugs are metabolized through the cytochrome P450 (CYP) enzymes CYP2D6, CYP3A4 [40], and CYP2C19 [42], drugs that decrease the activity of these enzymes can result in a rise to toxic serotonin levels over an unpredictable length of time. Similarly, because the monoamine oxidase system degrades serotonin to 5-hydroxyindoleacetic acid for excretion, ref. [40] Monoamine oxidase inhibitors are considered particularly likely causes of serotonin syndrome [43]. 

Examples of drugs commonly used in internal medicine that may contribute to the development of serotonin syndrome include:

Analgesics: Tramadol, Fentanyl
Antimigraine (Triptans)
Anticonvulsants: Valproic acid
Skeletal Muscle Relaxants: Cyclobenzaprine
Cough suppression: Dextromethorphan
Antinausea/Antiemetics: Ondansetron
Antibiotics: Linezolid [40]
Antihistamines: Chlorphenamine [43]

Treatment for serotonin syndrome includes discontinuation of all serotonergic drugs, administration of SERT receptor antagonists, such as cyproheptadine, supportive therapy, and sedation with benzodiazepines [41].

### 2.6. Persistent Pulmonary Hypertension in Newborns

Analysis of 143,591 pregnancies from the Quebec statistics database demonstrated a significant association between maternal use of SSRIs after week 21 of gestation and the occurrence of persistent pulmonary hypertension in newborns. No association was found for mothers taking SSRIs before week 21 of gestation [44].

### 2.7. Prolonged QT Interval

The occurrence of Torsades de Pointes, a form of polymorphic ventricular tachycardia characterized by a prolonged QT interval, is used as a proxy for the potential of medication to cause fatal arrhythmias. Although there is only a modest association between prolonged QT intervals and the actual occurrence of Torsades de Points, no superior clinical indicator exists. Complicating matters further, there is currently no consensus on what degree of QT prolongation should be considered clinically significant. QT interval prolongation is heterogeneous among SSRIs. The relationship between QT prolongation and SSRIs are as follows:

- No QT prolongation: fluoxetine and sertraline
- QT prolongation only on coadministration with other QT prolongers: paroxetine
- Potential risk of prolonged QT in monotherapy: citalopram (demonstrated) and escitalopram (potential)

The effects in citalopram and escitalopram, which are dose-dependent, among other SSRIs, are notable because they are considered first-line drugs due to their otherwise favorable side-effect profiles, low degree of drug interaction, and low cost. As a result, alternative drugs should be considered among patients experiencing acute coronary syndromes. Important considerations also include advanced age, which increases the risk for prolonged QT interval, and poor CYP2C19 metabolism, which may cause supratherapeutic exposures at therapeutic doses [45].

### 2.8. Sexual Side Effects

Studies have demonstrated that 90% of men and 96% of women report the experience of side effects in at least one area of sexual functioning, and 20%–50% experience sexual side effects sufficiently significant to seek clinical assistance [46]. Serotonin is a known inhibitor of sexual desire and physical arousal, and orgasm [47]. These effects are thought to be related to off-target activity at 5HT2 receptors in the brain, affecting psychological, sexual function, and in the spinal cord, affecting physical aspects of arousal [42]. Although experimental therapies have attempted to counteract these side effects, the complex interactions that occur during the sexual response cycle may result in successfully addressing a single facet of sexual dysfunction while failing to address others. In one example, Ahrold and Meston successfully redressed deficits in women’s genital arousal with the administration of ephedrine but did not directly increase mental, sexual arousal. As a result, the administration of ephedrine did not positively affect the perception of sexual pleasure in the short term [46].

### 2.9. Gastrointestinal Side Effects

Off-target stimulation of central and peripheral 5HT3 receptors may result in nausea with associated vomiting or diarrhea [42].

### 2.10. Sedation

Paroxetine alone among the SSRIs may cause sedation due to its unique antihistamine properties [42].

### 2.11. SSRI Induced Activation

SSRIs have also been implicated in the activation of hypomania in patients who have an underlying bipolar disorder. In a case report detailing seven patients, they found that hypomania was uncovered after administration of an SSRI, with paroxetine being one of the main offenders [48]. Insomnia can also result from SSRI activation [48].

### 2.12. SSRIs Pharmacokinetics/Pharmacodynamics

SSRIs have good bioavailability in the gastrointestinal tract, with peak serum concentrations achieved 5–8 h after oral administration. Bioavailability, which is partially mediated by the effects of first-pass metabolism, is generally good. For SSRIs with relatively low bioavailability (e.g., paroxetine ~50%), bioavailability may improve upon achieving dosages that result in saturation kinetics. High levels of protein-bound transport and large areas of distribution are observed due to the strong lipophilicity of SSRIs [49].

SSRIs undergo hepatic metabolism with a half-life of 18–24 h. Fluoxetine is exceptional in forming a demethylated norfluoxetine metabolite that is active for several days. Most SSRIs undergo hepatic metabolism by the P450 enzyme CYP2D6. Alternatively, citalopram and escitalopram are metabolized by CYP2C19, whose levels physiologically decrease with age, necessitating dosage decreases in the elderly [42]. Importantly, co-administration of other drugs that reduce the efficiency of these P450 enzymes can produce toxic levels of synaptic serotonin toxicity in the setting of SSRI therapy. Of note, the catechol intermediate formed by paroxetine is excreted mainly via a renal route [49].

## 3. Clozapine

Clozapine was regarded as an atypical when it was first discovered in 1959 because it was thought that extrapyramidal side effects were a hallmark of antipsychotic efficacy [50], but its efficacy was not based on the development of these symptoms [51]. However, development of the drug has been stymied due to reports of patients developing agranulocytosis [52,53]. Careful blood testing at regular intervals and cessation of therapy at the first sign of possible agranulocytosis are now part of the treatment protocols for clozapine [54,55]. A 2018 study by Munro et al. found Asian populations were as much as 2.4 times more likely to develop agranulocytosis [56]; Moeller et al. found that African-American patients were twice as likely to have treatment withdrawn because they had a significantly lower baseline WBC count than non-African American patients [56,57].

Clozapine remains the only effective drug against treatment-resistant schizophrenia, defined as failing treatment with at least two different antipsychotic medications [58]. In the clinical trial by Kane et al. patients who failed treatment with haloperidol for six weeks were then randomly assigned to a 6-week course of either clozapine or chlorpromazine [54]. Mean scores on the brief psychiatric rating scale (BPRS) and the clinical global impressions (CGI) scale were significantly higher for patients treated with clozapine over those treated with chlorpromazine, and as many as 30% of patients treated with clozapine showed a response to the drug [54]. However, its side effect profile (which, in addition to the aforementioned agranulocytosis, includes sedation, postural hypotension, gastrointestinal hypomotility, diabetes mellitus, dyslipidemia, and more serious events such as myocarditis and cardiomyopathy) remains a major cause for concern among clinicians [59].

Clozapine has been demonstrated to lower suicidal behavior amongst inpatients with schizophrenia. A retrospective study by Modestin et al. showed suicidal behavior rate to drop from 28% to just 3% while on clozapine, then regression to 18% following discontinuation of therapy [60]. One of its more unique effects, however, may lie in its ability to decrease substance abuse. In a randomized trial, clozapine was associated with decreased cannabis use [61]; a naturalistic study showed a reduction of alcohol use from 54.1 drinking days to 12.5 while on clozapine [62]. A definitive study on clozapine’s ability to reduce substance use, however, has yet to be performed [63].

### Clozapine Mechanism of Action, Pharmacokinetics, and Pharmacodynamics

Clozapine acts at type 2 and type 4 dopamine receptors, type 2 serotonin receptors, and receptors of norepinephrine, histamine, and acetylcholine [64]. The binding of D2 currently appears to be an obligate property of successful antipsychotic medications. Clozapine’s ability to bind weakly and transiently differs from strongly binding traditional antipsychotics like haloperidol. Transient binding of D2 receptors on the striatum confers antipsychotic properties while minimizing motor side-effects [65]. Whereas D2 receptors mediate locomotion, attention, sleep, memory, learning, D4 receptors mediate attention, sleep, memory, learning. Both D2 and D4 receptors act intracellularly by inhibiting adenylyl cyclase and activating potassium channels. While clozapine binds with greater affinity to D4 than to D2 receptors, it is notable that D4 is the least abundant class of dopamine receptors. Both receptors are expressed with greatest density in the striatum and are important for survival signaling in dopaminergic neurons [66].

Like other atypical antipsychotics, clozapine acts as a 5HT_2A_ serotonin receptor antagonist. 5HT_2A_ receptors are implicated in the pathogenesis of depression, schizophrenia, and addictive disorders. Additionally, many hallucinogenic drugs of abuse display 5HT_2A_ agonism [67]. The relatively high affinity of clozapine to 5HT_2A_ receptors is suspected to be the basis for its superior efficacy in treatment-resistant schizophrenia [68]. Moreover, while clozapine’s D2 antagonism relieves the positive symptoms of schizophrenia, 5-HT2A antagonism relieves its negative symptoms [69].

Like SSRIs, clozapine is extensively metabolized by the hepatic cytochrome P450 (CYP) system. Somewhat conversely to SSRIs, the major metabolizing enzymes for clozapine are CYP3A4 and CYP1A2, with CYP2D6 playing only a minor role in metabolism. The activation of CYP1A2 by cigarette abuse may lead to lower serum levels of clozapine than in nonsmokers. A notable inhibitor of CYP1A2 is the SSRI fluvoxamine. Also of note, the rate of clozapine metabolism is observed to fall with age, necessitating dosing adjustments. Clozapine has a half-life of 8 h, with elimination via hepatic and renal routes [70]. 

## 4. SSRIs and Augmentation

The antidepressant effects of an SSRI alone may prove insufficient to completely treat major depression in 29–46% of patients, necessitating the use of adjuvant pharmaceutical therapies [71]. One advantage of adjuvant pharmacotherapy is the avoidance of pharmacotherapy lag time that would otherwise be encountered when transitioning from one SSRI to another. Over time, potentially effective pharmaceutical adjuvants have included non-SSRI antidepressants, bupropion, lithium, triiodothyronine, and atypical antipsychotic medications [5]. The need for continuous clinical monitoring of levels of lithium and triiodothyronine has made them relatively unpopular as treatment choices for clinicians. Likewise, the narrow therapeutic applications of lithium and the potential side-effects of triiodothyronine in otherwise euthyroid patients limit the utility of these drugs as adjuvants to SSRIs [72].

The mechanism by which atypical antipsychotics enhance the action of SSRIs is unknown, and their addition may cause both increased efficacy or increased off-target effects [5]. Studies by Danovich et al. directly measured changes in the levels of GABA_A_ β2/3 receptor protein expression and phosphorylation levels in cultured cortical cells derived from rats, upon administration of either haloperidol plus fluvoxamine or clozapine alone. In experiments testing the combination of haloperidol and fluvoxamine, the administration of both drugs together showed the inducement of GAD67 and PKCβ2 genes associated with GABAergic transmission that were not activated by either of these drugs alone. Thus, in the case of haloperidol and fluvoxamine, augmentation therapy has shown the capacity to not only amplify the efficacy of pharmacotherapy but to exhibit novel therapeutic properties. These investigators noted that some changes affected by the combination of the haloperidol and fluvoxamine were similar to the cellular effects observed with the addition of clozapine alone [73]. Despite acknowledged limitations, this study is important because it elucidates at least one potential mechanism for the synergy between SSRIs and antipsychotic medications, which are largely unknown otherwise. 

Separately, the failure of some instances of SSRI monotherapy for major depression has been attributed to the SSRI’s moderate off-target suppression of norepinephrine levels, which are experimentally demonstrable. The demonstrated ability of aripiprazole to attenuate these off-target effects on norepinephrine is another putative mechanism for the effectiveness of SSRI augmentation [74,75].

While quetiapine and aripiprazole have shown the most efficacy for SSRI augmentation in treatment-resistant depression [72], olanzapine has demonstrated superior efficacy as an SSRI augmenter in treatment-resistant panic disorder [76].

Patients with schizophrenia who display a constellation of negative symptoms respond poorly to antipsychotic monotherapy. Negative symptoms include the diminution of motivation, interest, or expression. These symptoms are often responsible for a high proportion of morbidity in schizophrenia [77]. Conversely to the depressive symptomology of interest presently, supplementation of antipsychotics with SSRIs in schizophrenic patients with negative symptoms lead to synergistic improvements in the function of these patients. 

Second-generation antipsychotics (SGAs) are also associated with the development of hyponatremia. Clozapine is one of the most common SGAs associated with the development of hyponatremia [78]. The average drop in sodium levels was around 112 mmol/L and returned to normal levels once the SGA was stopped [78]. This is important to note this common association as hyponatremia is also a side effect of SSRI use [79]. Using both of these together could lead to severe hyponatremia, which should be monitored to prevent.

In summary, although the augmenting capabilities of atypical antipsychotics are clinically appreciated for the augmentation of SSRI therapies, our understanding of the exact mechanisms of augmentation remain in their infancy. However, where mechanisms have been partially elucidated, it appears that mechanism of actions for combination therapy are distinct from either drug in monotherapy. 

## 5. Drug Interactions with Clozapine Levels/Drug Interactions

The cytochrome P450 system plays a significant role in the metabolism of clozapine [80] and, thus, drug-drug interactions can result, and drug interactions can also affect clozapine levels in the body. CYP1A2 is the primary metabolizer of clozapine, which it converts to norclozapine; Bertilsson et al. demonstrated that N1- and N7-demethylation of caffeine correlated strongly with CYP1A2 activity and clozapine clearance [81]. Fluvoxamine is one of the most potent inhibitors of CYP1A2; a case report of a 44-year-old male patient showed that he exhibited up to 4160 mcg/L of plasma clozapine (normal range 200–600 mcg/L) after the addition of fluvoxamine [82]. Similar increases in serum clozapine were detected in two patients taking sertraline [83]. Smoking can also induce CYP1A2 and reduce serum clozapine levels [84]. Haring et al. described a 20% lower plasma concentration of clozapine in smokers when compared with non-smokers [85]; Wetzel et al. describes a similar decrease in smokers [86]. Subsequent studies observed a comparable decrease in plasma clozapine concentration in smokers to an increase in CYP1A2 activity measured by caffeine clearance [87,88]. The proton pump inhibitor omeprazole also induces CYP1A2 and reduced plasma levels of clozapine in two patients [89].

Clozapine can also be metabolized by CYP3A4 to clozapine-N-oxide, but this is thought to be a minor pathway [90]. Concomitant administration of erythromycin, an inhibitor of CYP3A4, caused clozapine toxicity and elevated serum levels in a patient [91]. A subsequent 1999 study found no significant differences in clozapine serum concentration upon erythromycin administration, though Hagg et al. acknowledge that this result may have been influenced by a short 9-h erythromycin pre-treatment period [92]. A similar lack of effect on clozapine concentration was also exhibited by itraconazole, another CYP3A4 inhibitor [93]. Nefazodone, another CYP3A4 inhibitor [94], showed only minimal increases on clozapine and norclozapine levels in a study of six patients [95]. Fluoxetine, a common SSRI used for depression, has been known to increase clozapine levels. There have been different studies that have reported differing amounts in the increase, but fluoxetine in these studies have shown to increase clozapine levels between 40% and 70% [96].

There is a relative lack of data amongst the literature where other CYPs are concerned. In vitro studies have shown that clozapine can inhibit CYP2C9, CYP2C19, CYP2D6, and CYP3A [97], with a possible preference for CYP2D6 [98]. Data on the possible interaction between clozapine and paroxetine, a potent inhibitor of CYP2D6, are somewhat contradictory [99]. Centorrino et al. reported a significant 56.6% serum clozapine level increase with concomitant paroxetine treatment [100], while Wetzel et al. reported paroxetine did not cause significant pharmacokinetic interactions at a dose of 20 mg/day [86].

Clozapine levels are also associated with infection-related inflammation, with a median increase of 48% in inflammation based on a CRP of 5 mg/L or higher [101,102]. One recent case study of a 46-year-old nonsmoker with schizophrenia examined the relationship between clozapine levels and COVID-19 infection; however, measurement of CRP failed during an episode where the patient’s serum clozapine level tripled in the presence of pneumonia and COVID-19 [103]. Another patient who had received the Pfizer-BioNTech vaccine for SARS-CoV-2 had his clozapine level double after he experienced adverse effects four days post-vaccination; these were later attributed to similar CYP1A2 inhibition mechanisms [104]. In any case, the literature recommends monitoring the clozapine plasma concentration in the presence of infectious symptoms or reducing the dose in half; the dosage can be resumed within three days of symptom resolution or CRP normalization [105].

## 6. Clinical Studies: Safety and Efficacy

A subset of patients will fail to respond or only partially respond to clozapine, leading clinicians to seek an additional medication for concomitant use with clozapine to improve treatment outcomes. Patients suffering from co-existent depression may benefit from adding an SSRI to their clozapine treatment regimen. Studies have investigated the safety and efficacy of clozapine combination therapies with various SSRIs, including fluvoxamine, paroxetine, sertraline, fluoxetine, and citalopram. 

Fluvoxamine is an SSRI that has been more extensively studied with clozapine compared to other SSRIs. Fluvoxamine is an inhibitor of cytochrome P450 (CYP450) isoenzymes, particularly CYP1A2 and, to a lesser degree, CYP2C19 and CYP3A4 [106,107,108,109]. CYP1A2 is the main isoform in clozapine metabolism, and thus concomitant use of clozapine with fluvoxamine may hinder clozapine metabolism and potentially cause adverse side effects. Many studies have been carried out to analyze the degree to which fluvoxamine increases plasma levels of clozapine and its metabolites, N-desmethylclozapine and clozapine N-oxide [110,111]. All studies investigating the effects of fluvoxamine, as described in Table 1, observed increased levels of clozapine, N-desmethylclozapine, and clozapine N-oxide following coadministration of fluvoxamine and clozapine [100,107,112]. 

Szegedi A et al. conducted a study in 1999 to examine the serum concentrations for clozapine and its metabolites (N-desmethylclozapine, clozapine N-oxide) before and after coadministration with fluvoxamine [107]. The study included a total of 16 patients suffering from schizophrenia with predominant negative symptomology (*n* = 15) or delusional disorder (*n* = 1). All patients were treated for a minimum of six weeks. Initial treatment involved a monotherapy with clozapine (2.5–3.5 mg/kg; 125–250 mg/day), followed by an add-on therapy of 50 mg of fluvoxamine. Increased serum concentrations of clozapine and its metabolites were observed following the addition of fluvoxamine to the treatment regimen (day 7: clozapine serum concentrations increased 2.3-fold, N-desmethylclozapine increased 2.1-fold; day 14: clozapine serum concentrations increased 2.6-fold, N-desmethylclozapine increased 2.6-fold). However, no significant changes in the frequency or severity of treatment-related adverse effects were reported despite the increase in clozapine serum concentrations. 

The study went on to conclude that the concomitant use of clozapine and fluvoxamine does, in fact, cause an increase in circulating concentrations of clozapine and N-desmethylclozapine and clozapine N-oxide, as predicted [107]. However, coadministration of the two drugs was well-tolerated by patients, causing no apparent critical side effects despite the increased clozapine, N-desmethylclozapine, and clozapine N-oxide serum concentrations. This finding was inconsistent with previous studies that reported more pronounced changes in laboratory and ECG parameters with increased serum clozapine levels [107,113,114]. This inconsistency may support a current hypothesis that increasing clozapine dosage and the addition of fluvoxamine do not have the same effects on the patient, despite both causing an increase in circulating clozapine concentrations. Psychopathology improvement was observed in clozapine monotherapy and continued after the addition of fluvoxamine. Overall, findings suggest that a combination treatment of clozapine with fluvoxamine can be an effective treatment option for patients suffering from schizophrenia with co-existent depression but should only do so if the patient’s clozapine serum concentrations are being closely monitored.

Another study investigating the therapeutic efficacy of clozapine and fluvoxamine coadministration looked specifically at the effects of fluvoxamine on clozapine-related weight gain, hyperglycemia, and lipid abnormalities [112]. Treatment-resistant inpatients with a DSM-IV schizophrenia diagnosis (*n* = 68) were randomly assigned to 2 treatment groups for 12 weeks; monotherapy group (*n* = 34) received clozapine (≤600 mg/day), coadministration group (*n* = 34) received fluvoxamine (50 mg/day) plus low-dose clozapine (≤250 mg/day). The monotherapy group showed a significant increase (*p* < 0.05) in body weight, BMI, and serum glucose after treatment compared to baseline. Bodyweight, BMI, and serum glucose also increased in the coadministration group; however, none were found to be statistically significant. At week 12, the monotherapy group had significantly higher glucose (*p* = 0.035), triglyceride (*p* = 0.041), and norclozapine (*p* = 0.009) compared to the coadministration treatment group. Overall, the results from the study provided evidence that the concomitant use of clozapine and fluvoxamine can attenuate weight gain and metabolic disturbances. Additionally, plasma levels of the metabolite norclozapine were specifically found to be associated with increases in weight, serum glucose, and triglyceride levels; circulating clozapine levels were not associated with these parameters.

One study investigated the therapeutic efficacy of adding citalopram to a clozapine treatment regimen in patients with schizophrenia by identifying potential drug interactions that would hinder the metabolism of clozapine. This was a preliminary study that used psychiatric unit inpatients receiving clozapine treatment for refractory schizophrenia but still suffered from co-existent depression that necessitated treatment (*n* = 5). Participants received an initial treatment of clozapine monotherapy that was administered at a constant dose (500–600 mg/day) for two weeks. All baseline clozapine plasma levels were obtained and recorded. Citalopram (20 mg/day) was then added to the treatment regimen for a minimum of two weeks. Plasma clozapine levels were obtained on day seven and day 14 of concomitant drug use. Samples were routinely taken 12 h after the night-time dose of clozapine. Clozapine concentration values were determined via high-performance liquid chromatography. Data obtained from the coadministration of citalopram with clozapine were as follows: plasma clozapine levels decreased in three patients (patients 1, 3, 5), increased in one patient (patient 2), and remained unchanged in one patient (patient 4). The overall trend from these data suggest citalopram generally causes a decrease in plasma clozapine and norclozapine levels. Citalopram did not appear to affect the relationship between clozapine and its metabolites as the study found the ratio of clozapine to norclozapine plasma levels remained much unchanged. This suggests that citalopram does not appear to have any obvious effects on clozapine metabolism, which is an important distinction from other SSRIs that do interfere with the metabolism of clozapine. The cause for fluctuations in clozapine plasma levels was unclear, but the study attributed this to most likely be due to the variations in absorption, distribution, and metabolism unique to each participant. Based on the limited evidence obtained from the preliminary study, citalopram appears to potentially be the SSRI of choice in those taking clozapine. However, a larger study must be carried out to increase the statistical significance of the results.

Spina E. et al. conducted a study to determine the therapeutic efficacy of two SSRIs, paroxetine and sertraline, when added to a clozapine treatment regimen in patients with schizophrenia [115]. The study was designed as an open pharmacokinetic investigation and observation were limited to the first three weeks of coadministration of clozapine with paroxetine or sertraline. Seventeen outpatients were selected to participate, 11 males and six females (*n* = 17), ranging in age from 29 to 55 years old, and had been previously given a DSM IV diagnosis of schizophrenia (*n* = 13) or schizoaffective disorder (*n* = 4). All participants suffered from co-existent depression that necessitated treatment with an SSRI. Patients were initially treated with a constant dose of clozapine for a minimum of three months; clozapine dose ranged from 200 to 400 mg/day between patients. Following the establishment of steady-state clozapine levels, one of the two SSRIs was added to the daily treatment regimen; paroxetine (20–40 mg/day; *n* = 9) or sertraline (50–100 mg/day; *n* = 8). Doses for all medications were kept constant throughout the entire study period. High-performance liquid chromatography (HPLC) was used to determine the steady-state plasma concentrations of clozapine, norclozapine, and clozapine N-oxide, according to Avenoso et al. [116]. 

The results from the study showed the addition of paroxetine at 20 to 40 mg/day to patients stabilized on clozapine therapy resulted in a significant elevation in the serum concentrations of clozapine. Paroxetine also appeared to cause an increase in the concentration of the clozapine metabolites, norclozapine and clozapine N-oxide. Norclozapine plasma levels significantly increased (*p* < 0.05), while clozapine N-oxide levels only showed a slight increase which was not statistically significant. Paroxetine is known to be a potent inhibitor of CYP2D6, which plays a key role in clozapine metabolism. Investigators believe the inhibitory effects of paroxetine seem to be mediated or modulated via pathways other than N-demethylation and N-oxidation. After three weeks of coadministration with paroxetine, steady-state plasma levels for clozapine and norclozapine were an average of 31% and 20% higher, respectively, compared to baseline levels. This paroxetine-induced elevation of circulating clozapine levels was not associated with enhanced efficacy or clinically linked adverse events. Additionally, despite this increase in plasma levels, paroxetine did not alter the ratios between clozapine and its metabolites, which remained consistent throughout the investigation. In contrast, sertraline was not found to significantly modify mean plasma concentrations of clozapine and its metabolites, causing an 11% increase in clozapine plasma levels and a 5% increase in norclozapine. This drug combination was also well-tolerated by participants, demonstrating a rise in clozapine plasma levels was not correlated to a rise in adverse side effects. In summary, the findings from this study suggest sertraline may be an acceptable treatment option for patients taking clozapine, but strongly advise when paroxetine is co-administered with clozapine that clinicians provide patients with careful clinical observation and monitoring. Caution with concomitant use of paroxetine is particularly important as the inhibitory effect of paroxetine on CYP enzymes is concentration-dependent, and this relationship may be amplified at higher doses due to its non-linear pharmacokinetics [117,118].

Centorrino, F et al. compared the therapeutic efficacy of three different SSRIs, fluoxetine, paroxetine, and sertraline, when added to a clozapine treatment regimen for patients suffering from schizophrenia [100]. The main objective of the study was to identify any potential drug interactions between the SSRIs and clozapine that would hinder the metabolism of clozapine. Such interactions are of interest because of the limited tolerability of high doses and circulating concentrations of clozapine [119]. These interactions with clozapine likely reflect the antagonistic interactions of SSRIs with the genetically complex hepatic cytochrome P450 oxidase isozymes that play key roles in metabolizing exogenous compounds [120,121,122,123]. Fluoxetine interacts with CYP2D6, CYP2C19, and CYP3A3-4; sertraline also interacts with these same CYP isozymes plus CYP1A2; and paroxetine mainly interacts with CYP2D6 but may also potentially interact with CYP3A3-4 [120,121,123,124,125]. This study assessed serum concentrations of clozapine and norclozapine in 80 psychiatric patients, matching for age and clozapine dose. The patients received either clozapine alone (mean dose = 279 mg/day) or in combination with one of the three SSRIs: fluoxetine (mean dose = 39.3 mg/day), paroxetine (mean dose = 31.2 mg/day), or sertraline (mean dose = 92.5 mg/day). Each patient’s clozapine dose was stable for a minimum of 1 month before serum sampling. The serum concentrations of clozapine and norclozapine were obtained using liquid chromatography and computerized ultraviolet spectrophotometry. In patients taking SSRIs, the sum of clozapine and norclozapine plasma levels averaged approximately 43% higher than patients taking clozapine alone. Findings also suggest the risk of clozapine levels higher than 1000 ng/mL was 10-fold greater (25%) in patients taking any of the 3 SSRIs, with minor differences between the individual SSRIs, indicating these SSRIs can increase circulating levels of clozapine and norclozapine to potentially toxic levels. In conclusion, the study recommended patients be monitored closely when taking clozapine in combination with an SSRI, especially when the daily clozapine dose exceeds 300 mg or 3/5 mg/kg.

Spina et al. looked at fluoxetine and its effects on the plasma concentrations of clozapine and its major metabolites. Patients were stabilized on clozapine, 200–450 mg/day, and then received 20 mg/day of fluoxetine for eight consecutive weeks. The mean plasma concentrations of clozapine were increased significantly by 58%, norclozapine by 36% and clozapine N-oxide by 38%. The drug combination was well tolerated. They concluded that clozapine levels should be monitored if fluoxetine is started [126]. However, a fatal drug interaction was noted in a case report between clozapine and fluoxetine. The decreased in this case report had toxicity levels of both clozapine and fluoxetine in his blood. The deceased also had pulmonary edema, visceral vascular congestion, paralytic ileus, gastroenteritis, and eosinophila, which are associated with clozapine toxicity [127]. This highlights the importance of monitoring of drug levels when using these two medications.

Rahman, MS et al. discussed a case report involving the treatment of clozapine-induced obsessive-compulsive disorder (OCD) with sertraline, taking advantage of sertraline’s lack of liver enzyme competition [128,129]. The patient in the case report was a 39-year-old white male with a 20-year history of paranoid schizophrenia. He had been previously treated with clozapine, which significantly improved his psychotic symptoms, but he, unfortunately, developed severe clozapine-induced OCD behaviors two years into treatment and was forced to discontinue the clozapine therapy. Other treatment options were not effective for the patient. Due to a lack of additional treatment options, the patient was restarted on a clozapine treatment regimen, but this time with the addition of an SSRI to avoid a return of OCD behaviors. The initial SSRI used for the patient’s OCD treatment was fluvoxamine (50 mg/day). The patient’s OCD behavior did improve with fluvoxamine (150 mg/day). However, this SSRI caused clozapine plasma levels to rise to potentially toxic amounts over 700 ng/mL, and the patient responded poorly to medication adjustments to account for this fluvoxamine-induced increase (325 mg/day clozapine, 50 mg/day fluvoxamine). Fluvoxamine was then replaced with sertraline (100 mg twice daily). Sertraline proved to be more effective compared to fluvoxamine. The patient’s psychotic symptoms and obsessive-compulsive behaviors were well controlled by the combination. Sertraline also did not affect clozapine plasma levels or its efficacy. In conclusion, this case report demonstrated that SSRIs can effectively treat clozapine-induced ODC behaviors. Comparison of the two SSRIs showed fluvoxamine was effective in treating the clozapine-induced OCD behavior but negatively interfered with clozapine metabolism and, as a result, significantly increased clozapine plasma levels to potentially toxic levels. Sertraline was selected as the preferred SSRI of choice for the treatment of clozapine-induced OCD behavior because it proved to be both effective and did not cause a significant increase in clozapine plasma levels.

## 7. Conclusions

SSRIs have historically improved upon the mechanisms of action and consequent side effect profiles of tricyclic antidepressants and have become the first-line treatment of choice for major depression. Nonetheless, SSRI monotherapy fails to resolve depression in a significant number of patients. Atypical antipsychotics, such as aripiprazole, quetiapine, ziprasidone, olanzapine, and clozapine, have shown efficacy in improving the effectiveness of SSRIs in the treatment of major depression, panic disorder, obsessive-compulsive disorder, and other psychiatric conditions that prove refractory to first-line SSRI therapy. Although little is known about the mechanisms of action in these augmented therapies, some studies have shown not only augmentation of first-line mechanisms but also the activation of novel mechanisms that are not activated by either drug in monotherapy. 

Clozapine, although carrying a particularly onerous burden of serious off-target effects, shows unique efficacy in the treatment of treatment-resistant depression. The substantial risks of clozapine’s potentially permanent and deleterious off-target effects are balanced in some patients by the need to control the otherwise unacceptable risk of suicidality in some patients. Although our understanding of the mechanisms these mechanisms of action for combinatorial therapy with SSRIs and atypical antipsychotics remains in its infancy, the potential for the discovery and exploitation of novel therapeutic mechanisms appears promising and warrants further scrutiny. Initial in vitro studies on the combinatorial mechanisms of fluvoxamine and haloperidol have demonstrated mechanical similarities to clozapine. Given clozapine’s extreme off-target effects, one direction for further study may be in vivo trials of such combinatorial therapies as a superior alternative to clozapine as mechanism of augmentation, although the risks of adding fluvoxamine to another SSRI warrant monitoring for the development of serotonin syndrome. 

## Figures and Tables

**Table 1:** Clinical studies combining clozapine with SSRIs.

Author (Year)	Drugs Studied/Objective	Groups Studied and Intervention	Results and Findings	Conclusions
Szegedi A. et al. (1999) [107]	To determine if the concomitant use of clozapine + fluvoxamine (SSRI) improves therapeutic efficacy in schizophrenic patients who experience negative symptoms with clozapine.	The study examined serum concentrations for clozapine and coadministration of fluvoxamine. Patients were treated for 6 weeks or more. Initial treatment was a monotherapy with clozapine (2.5–3.5 mg/kg; 125–250 mg/day), followed with an add-on therapy of 50 mg of fluvoxamine.	Increase in serum concentrations of clozapine and its metabolites observed following addition of fluvoxamine to treatment regimen (Day 7: clozapine serum concentrations increased 2.3-fold, N-desmethylclozapine increased 2.1-fold; Day 14: clozapine serum concentrations increased 2.6-fold, N-desmethylclozapine increased 2.6-fold). No significant changes in adverse effects were reported (frequency, severity) despite increased clozapine serum concentrations with concomitant drug administration.	Concomitant use of clozapine and fluvoxamine was well tolerated, critical side effects absent. Psychopathology improvement observed in clozapine monotherapy and continued after addition of fluvoxamine. Combined treatment should only be prescribed under close monitoring of clozapine serum concentrations.
Mong-Liang L et al. (2004) [112]	To demonstrate the effects of fluvoxamine (SSRI) on clozapine-related weight gain, hyperglycemia, and lipid abnormalities.	Treatment-resistant inpatients with a DSM-IV schizophrenia diagnosis (*n* = 68) randomly assigned to 2 treatment groups for 12 weeks; monotherapy group (*n* = 34) received clozapine (≤600 mg/day), coadministration group (*n* = 34) received fluvoxamine (50 mg/day) plus low-dose clozapine (≤250 mg/day).	Monotherapy group showed significant increase (*p* < 0.5) in body weight, BMI, serum glucose after treatment than at baseline; increase also seen in coadministration group but not statistically significant. At week 12, monotherapy group had significantly higher glucose (*p* = 0.035), triglyceride (*p* = 0.041), and norclozapine (*p* = 0.009) compared to coadministration group.	Results suggest concomitant use of clozapine and fluvoxamine can attenuate weight gain and metabolic disturbances. Plasma levels of norclozapine, but not clozapine, found to be associated with increases in weight, serum glucose, and triglyceride levels.
Taylor D et al. (1997) [130]	To determine the therapeutic efficacy of adding citalopram (SSRI, antidepressant) to a clozapine treatment regimen in patients with schizophrenia	Initial treatment: monotherapy of clozapine administered at constant dose (500–600 mg/day) for 2 weeks; baseline clozapine plasma levels recorded. Citalopram (20 mg/day) was then added to the treatment regimen for a minimum of 2 weeks. Plasma clozapine levels were obtained on day 7 and day 14 of concomitant drug use. Samples drawn 12 h. after night-time dose of clozapine.	Clozapine plasma levels increased in only one participant (patient 2). Plasma levels remained unchanged in patient 4 but decreased in the remaining three patients (patients 1, 3, 5). Overall, small decreases in plasma clozapine and norclozapine levels observed; ratio of clozapine:norclozapine remained much the same.	Citalopram had no clear effect on clozapine metabolism; important distinction from other SSRI’s that do interfere with clozapine metabolism. Based on limit evidence, study finds citalopram to be the potential SSRI of choice in those taking clozapine. Larger study needed to increase significance of results.
Spina. E et al. (2000) [115]	To determine the therapeutic efficacy of 2 SSRIs, paroxetine and sertraline, when added to a clozapine treatment regimen in patients with schizophrenia, ensuring no drug interactions exist that would hinder the metabolism of clozapine.	17 outpatients selected: 11 males 6 females (*n* = 17), ages 29–55 years old, and given DSM IV diagnosis of schizophrenia (*n* = 13) or schizoaffective disorder (*n* = 4). Patients treated with constant dose of clozapine for minimum of 3 months, then either 20–40 mg/day of paroxetine (*n* = 9) or 50–100 mg/day of sertraline (*n* = 8) was added to daily treatment regimen. Steady-state plasma concentrations of clozapine, norclozapine and clozapine N-oxide assayed by HPLC.	Paroxetine found to cause a significant increase in clozapine plasma levels (*p* < 0.01): baseline = 337 ± 83 ng/mL, week 3 = 441 ± 141 ng/mL. Paroxetine also found to significantly increase norclozapine plasma levels (*p* < 0.05). Paroxetine did not, however, alter ratios between clozapine and its metabolites. On the other hand, sertraline did not significantly modify mean plasma concentrations of clozapine and its metabolites.	Clozapine coadministration with paroxetine or sertraline was well tolerated. Findings suggest metabolism of clozapine is not affected by sertraline, while paroxetine, a potent inhibitor of CYP2D6, does have an effect and appears to inhibit metabolism of clozapine. While sertraline may be safely added to patients on maintenance treatment with clozapine, careful clinical observation and monitoring of a patient is recommended when paroxetine is co-administered with clozapine.
Centorrino, F et al. (1996) [100]	To determine the therapeutic efficacy of 3 SSRIs, fluvoxamine, paroxetine, and sertraline, when added to a clozapine treatment regimen in patients with schizophrenia, ensuring no drug interactions exist that would hinder the metabolism of clozapine.	Study assessed serum concentrations of clozapine and norclozapine in 80 psychiatric patients, matched for age and clozapine dose, given clozapine alone (mean dose = 279 mg/day) or in combination with one of the three SSRIs: fluoxetine (mean dose = 39.3 mg/day), paroxetine (mean dose = 31.2 mg/day), or sertraline (mean dose = 92.5 mg/day).	In patients taking SSRIs, the sum of clozapine and norclozapine plasma levels averaged approximately 43% higher than patients taking only clozapine. Findings also suggest the risk of levels higher than 1000 ng/mL was 10-fold greater (25%), in patients taking any of the 3 SSRIs, with minor differences between the individual SSRIs.	SSRIs can increase circulating levels of clozapine and norclozapine to potentially toxic levels. Patients should be monitored closely when taking clozapine in combination with an SSRI, particularly especially when the daily clozapine dose exceeds 300 mg or 3/5 mg/kg.
Rahman, MS et al. (1998) [128]	To review a case report describing treatment of clozapine-induced obsessive-compulsive disorder (OCD) with sertraline, which avoids liver enzyme competition.	Patient was a 39-year-old white male with a 20-year history of paranoid schizophrenia; previous clozapine treatment regimen significantly improved psychotic symptoms, but patient developed severe OCD behavior 2 years into treatment and forced to discontinue clozapine therapy. Initial treatment (325 mg/day clozapine, 50 mg/day fluvoxamine). Fluvoxamine discontinued, substituted for sertraline (100 mg b.i.d.) and raised clozapine to 475 mg/day.	The patient’s OCD behavior did improve with fluvoxamine (150 mg/day), however, the SSRI caused clozapine plasma levels to rise over 700 ng/mL. Fluvoxamine was replaced with sertraline (100 mg b.i.d.). Sertraline was more effective compared to fluvoxamine and patient’s psychotic and obsessive-compulsive symptoms were well controlled. Sertraline did not affect clozapine plasma level or efficacy.	SSRIs can effectively treat clozapine-induced ODC behaviors. Fluvoxamine was effective in treating OCD but caused significant increases in clozapine plasma levels. Sertraline was also found to be effective and did not cause significant increases in clozapine plasma levels.

## Data Availability

All data mentioned in this manuscript can be found on pub med.

## References

[1] Zhou J. (2004). Norepinephrine transporter inhibitors and their therapeutic potential. Drugs Future.

[2] Hovde M.J., Larson G.H., Vaughan R.A., Foster J.D. (2019). Model systems for analysis of dopamine transporter function and regulation. Neurochem. Int..

[3] Aggarwal S., Mortensen O.V. (2017). Overview of Monoamine Transporters. Curr. Protoc. Pharmacol..

[4] Joshi A. (2018). Selective Serotonin Re-uptake Inhibitors: An overview. Psychiatr. Danub..

[5] Wright B.M., Eiland E.H., Lorenz R. (2013). Augmentation with Atypical Antipsychotics for Depression: A Review of Evidence-Based Support from the Medical Literature. Pharmacother. J. Hum. Pharmacol. Drug Ther..

[6] Schildkraut J.J. (1965). The catecholamine hypothesis of affective disorders: A review of supporting evidence. Am. J. Psychiatry.

[7] Carlsson A., Biogenic A., Himwich H.E., Himwich W.A. (1964). Functional Significance of Drug-Induced Changes in Brain Monoamine Levels. Progress in Brain Research.

[8] Glowinski J., Kopin I.J., Axelrod J. (1965). Metabolism of [3 h] norepinephrine in the Rat Brain. J. Neurochem..

[9] Herting G., Axelrod J., Whitby L.G. (1961). Effect of drugs on the uptake and metabolism of H3-norepinephrine. J. Pharmacol. Exp. Ther..

[10] Feighner J.P. (1999). Mechanism of action of antidepressant medications. J. Clin. Psychiatry.

[11] Gardner D.M., Shulman K.I., Walker S.E., Tailor S.A. (1996). The making of a user friendly MAOI diet. J. Clin. Psychiatry.

[12] Shulman K.I., Walker S.E., MacKenzie S., Knowles S. (1989). Dietary restriction, tyramine, and the use of monoamine oxidase inhibitors. J. Clin. Psychopharmacol..

[13] Brambilla P., Cipriani A., Hotopf M., Barbui C. (2005). Side-effect profile of fluoxetine in comparison with other SSRIs, tricyclic and newer antidepressants: A meta-analysis of clinical trial data. Pharmacopsychiatry.

[14] Anderson I.M., Tomenson B.M. (1995). Treatment discontinuation with selective serotonin reuptake inhibitors compared with tricyclic antidepressants: A meta-analysis. BMJ.

[15] Weilburg J.B. (2004). An overview of SSRI and SNRI therapies for depression. Manag. Care.

[16] Compton S.N., Grant P.J., Chrisman A.K., Gammon P.J., Brown V.L., March J.S. (2001). Sertraline in Children and Adolescents With Social Anxiety Disorder: An Open Trial. J. Am. Acad. Child Adolesc. Psychiatry.

[17] Salaminios G., Duffy L., Ades A., Araya R., Button K.S., Churchill R., Croudace T., Derrick C., Dixon P., Dowrick C. (2017). A randomised controlled trial assessing the severity and duration of depressive symptoms associated with a clinically significant response to sertraline versus placebo, in people presenting to primary care with depression (PANDA trial): Study protocol for a randomised controlled trial. Trials.

[18] Lewis G., Duffy L., Ades T., Amos R., Araya R., Brabyn S., Button K.S., Churchill R., Derrick C., Dowrick C. (2019). The clinical effectiveness of sertraline in primary care and the role of depression severity and duration (PANDA): A pragmatic, double-blind, placebo-controlled randomised trial. Lancet Psychiatry.

[19] Treatment of Severe Obsessive-Compulsive Disorder with Fluvoxamine|American Journal of Psychiatry. https://ajp.psychiatryonline.org/doi/10.1176/ajp.144.8.1059?url_ver=Z39.88-2003&rfr_id=ori%3Arid%3Acrossref.org&rfr_dat=cr_pub++0pubmed&.

[20] Price L.H., Goodman W.K., Charney D.S., Rasmussen S.A., Heninger G.R. (1987). Treatment of severe obsessive-compulsive disorder with fluvoxamine. Am. J. Psychiatry.

[21] Gorman J.M., Kent J.M. SSRIs and SNRIs: Broad Spectrum of Efficacy Beyond Major Depression. https://www.psychiatrist.com/jcp/depression/ssris-snris-broad-spectrum-efficacy-beyond-major-depression/.

[22] Pigott T.A., Pato M.T., Bernstein S.E., Grover G.N., Hill J.L., Tolliver T.J., Murphy D.L. (1990). Controlled comparisons of clomipramine and fluoxetine in the treatment of obsessive-compulsive disorder. Behavioral and biological results. Arch. Gen. Psychiatry.

[23] Jenike M.A. (2004). Clinical practice. Obsessive-compulsive disorder. N. Engl. J. Med..

[24] Dunbar G., Steiner M., Bushnell W.D., Gergel I., Wheadon D.E. (1995). Long-term treatment and prevention of relapse of obsessive compulsive disorder with paroxetine. Eur. Neuropsychopharmacol..

[25] Bruce S.E., Goisman R.M., Salzman C., Spencer M., Machan J.T., Vasile R.G., Keller M.B. (2003). Are benzodiazepines still the medication of choice for patients with panic disorder with or without agoraphobia?. Am. J. Psychiatry.

[26] Tan J.Y., Levin G.M. (1999). Citalopram in the treatment of depression and other potential uses in psychiatry. Pharmacotherapy.

[27] Humble M., Wistedt B. (1992). Serotonin, panic disorder and agoraphobia: Short-term and long-term efficacy of citalopram in panic disorders. Int. Clin. Psychopharmacol..

[28] Stahl S.M., Gergel I., Li D. (2003). Escitalopram in the treatment of panic disorder: A randomized, double-blind, placebo-controlled trial. J. Clin. Psychiatry.

[29] Lepola U.M., Loft H., Reines E.H. (2003). Escitalopram (10–20 mg/day) is effective and well tolerated in a placebo-controlled study in depression in primary care. Int. Clin. Psychopharmacol..

[30] vanApeldoorn F.J., Stant A.D., van Hout W.J.P.J., Mersch P.P.A., den Boer J.A. (2014). Cost-effectiveness of CBT, SSRI, and CBT+SSRI in the treatment for panic disorder. Acta Psychiatr. Scand..

[31] Qi W., Gevonden M., Shalev A. (2017). Efficacy and Tolerability of High-dose Escitalopram in Posttraumatic Stress Disorder. J. Clin. Psychopharmacol..

[32] Barnett S.D., Tharwani H.M., Hertzberg M.A., Sutherland S.M., Connor K.M., Davidson J.R.T. (2002). Tolerability of fluoxetine in posttraumatic stress disorder. Prog. Neuropsychopharmacol. Biol. Psychiatry.

[33] Goldstein D.J., Wilson M.G., Thompson V.L., Potvin J.H., Rampey A.H. (1995). Long-term fluoxetine treatment of bulimia nervosa. Fluoxetine Bulimia Nervosa Research Group. Br. J. Psychiatry J. Ment. Sci..

[34] Walsh B.T., Wilson G.T., Loeb K.L., Devlin M.J., Pike K.M., Roose S.P., Fleiss J., Waternaux C. (1997). Medication and psychotherapy in the treatment of bulimia nervosa. Am. J. Psychiatry.

[35] Walsh B.T., Fairburn C.G., Mickley D., Sysko R., Parides M.K. (2004). Treatment of bulimia nervosa in a primary care setting. Am. J. Psychiatry.

[36] Milano W., Siano C., Putrella C., Capasso A. (2005). Treatment of bulimia nervosa with fluvoxamine: A randomized controlled trial. Adv. Ther..

[37] Milano W., Petrella C., Sabatino C., Capasso A. (2004). Treatment of bulimia nervosa with sertraline: A randomized controlled trial. Adv. Ther..

[38] Blier P., Szabo S.T. (2005). Potential mechanisms of action of atypical antipsychotic medications in treatment-resistant depression and anxiety. J. Clin. Psychiatry.

[39] Sprouse J., Braselton J., Reynolds L., Clarke T., Rollema H. (2001). Activation of postsynaptic 5-HT(1A) receptors by fluoxetine despite the loss of firing-dependent serotonergic input: Electrophysiological and neurochemical studies. Synapse.

[40] Beakley B.D., Kaye A.M., Kaye A.D. (2015). Tramadol, Pharmacology, Side Effects, and Serotonin Syndrome: A Review. Pain Physician.

[41] Lommel K.M., Meadows A.L., Chopra N., Thompson S., Stone C.K., Humphries R.L. (2017). Psychiatric Emergencies. CURRENT Diagnosis & Treatment: Emergency Medicine.

[42] O’Donnell J.M., Bies R.R., Shelton R.C., Brunton L.L., Hilal-Dandan R., Knollmann B.C. (2017). Drug Therapy of Depression and Anxiety Disorders. Goodman & Gilman’s: The Pharmacological Basis of Therapeutics.

[43] Scotton W.J., Hill L.J., Williams A.C., Barnes N.M. (2019). Serotonin Syndrome: Pathophysiology, Clinical Features, Management, and Potential Future Directions. Int. J. Tryptophan Res..

[44] Bérard A., Sheehy O., Zhao J.-P., Vinet É., Bernatsky S., Abrahamowicz M. (2017). SSRI and SNRI use during pregnancy and the risk of persistent pulmonary hypertension of the newborn. Br. J. Clin. Pharmacol..

[45] Funk K.A., Bostwick J.R. (2013). A comparison of the risk of QT prolongation among SSRIs. Ann. Pharmacother..

[46] Ahrold T.K., Meston C.M. (2009). Effects of SNS Activation on SSRI-Induced Sexual Side Effects Differ by SSRI. J. Sex Marital. Ther..

[47] Strachan D.A., O’Connell M.B., Smith J.A. (2019). Sexual Disorders. Women’s Health Across the Lifespan.

[48] Tanaka T., Inoue T., Suzuki K., Kitaichi Y., Masui T., Denda K., Koyama T. (2007). Clinical relevance of antidepressant-induced activation syndrome: From a perspective of bipolar spectrum disorder. Seishin Shinkeigaku Zasshi.

[49] van Harten J. (1993). Clinical pharmacokinetics of selective serotonin reuptake inhibitors. Clin. Pharmacokinet..

[50] Burns M.J. (2001). The pharmacology and toxicology of atypical antipsychotic agents. J. Toxicol. Clin. Toxicol..

[51] Stille G., Lauener H., Eichenberger E. (1971). The pharmacology of 8-chloro-11-(4-methyl-1-piperazinyl)-5H-dibenzo(b,e)(1,4)diazepine (clozapine). Il Farm. Ed. Prat..

[52] de la Chapelle A., Kari C., Nurminen M., Hernberg S. (1977). Clozapine-induced agranulocytosis. A genetic and epidemiologic study. Hum. Genet..

[53] Naheed M., Green B. (2001). Focus on clozapine. Curr. Med. Res. Opin..

[54] Kane J., Honigfeld G., Singer J., Meltzer H. (1988). Clozapine for the Treatment-Resistant Schizophrenic: A Double-blind Comparison With Chlorpromazine. Arch. Gen. Psychiatry.

[55] Mijovic A., MacCabe J.H. (2020). Clozapine-induced agranulocytosis. Ann. Hematol..

[56] Munro J., O’Sullivan D., Andrews C., Arana A., Mortimer A., Kerwin R. (1999). Active monitoring of 12760 clozapine recipients in the UK and Ireland: Beyond pharmacovigilance. Br. J. Psychiatry.

[57] Moeller F., Chen Y.-W., Steinberg J., Petty F., Ripper G., Shah N., Garver D. (1995). Risk Factors for Clozapine Discontinuation Among 805 Patients in the VA Hospital System. Ann. Clin. Psychiatry.

[58] Conley R.R., Kelly D.L. (2001). Management of treatment resistance in schizophrenia. Biol. Psychiatry.

[59] Flanagan R.J., Lally J., Gee S., Lyon R., Every-Palmer S. (2020). Clozapine in the treatment of refractory schizophrenia: A practical guide for healthcare professionals. Br. Med Bull..

[60] Modestin J., Dal Pian D., Agarwalla P. (2005). Clozapine diminishes suicidal behavior: A retrospective evaluation of clinical records. J. Clin. Psychiatry.

[61] Brunette M.F., Akerman S.C., Dawson R., O’Keefe C.D., Green A.I. (2016). An open-label pilot study of quetiapine plus mirtazapine for heavy drinkers with alcohol use disorder. Alcohol.

[62] Drake R.E., Xie H., McHugo G.J., Green A.I. (2000). The effects of clozapine on alcohol and drug use disorders among patients with schizophrenia. Schizophr. Bull..

[63] Khokhar J.Y., Henricks A.M., Kirk E., Green A.I. (2018). Unique Effects of Clozapine: A Pharmacological Perspective. Adv. Pharmacol..

[64] National Library of Medicine (US) (2004). National Center for Biotechnology Information. PubChem Compound Summary for CID 135398737, Clozapine. http://pubchem.ncbi.nlm.nih.gov/compound/Clozapine.

[65] Seeman P. (2014). Clozapine, a fast-off-D2 antipsychotic. ACS Chem. Neurosci..

[66] Bhatia A., Lenchner J.R., Saadabadi A. (2021). Biochemistry, Dopamine Receptors.

[67] Zhang G., Stackman R.W. (2015). The role of serotonin 5-HT2A receptors in memory and cognition. Front. Pharmacol..

[68] Schmidt C.J., Sorensen S.M., Kehne J.H., Carr A.A., Palfreyman M.G. (1995). The role of 5-HT2A receptors in antipsychotic activity. Life Sci..

[69] Wishart D.S., Feunang Y.D., Guo A.C., Lo E.J., Marcu A., Grant J.R., Sajed T., Johnson D., Li C., Sayeeda Z. (2018). DrugBank 5.0: A Major Update to the DrugBank Database for 2018. Nucleic Acids Res..

[70] Thorn C.F., Müller D.J., Altman R.B., Klein T.E. (2018). PharmGKB summary: Clozapine pathway, pharmacokinetics. Pharmacogenet. Genom..

[71] Papakostas G.I., Petersen T.J., Nierenberg A.A., Murakami J.L., Alpert J.E., Rosenbaum J.F., Fava M. (2004). Ziprasidone augmentation of selective serotonin reuptake inhibitors (SSRIs) for SSRI-resistant major depressive disorder. J. Clin. Psychiatry.

[72] Zhou X., Ravindran A.V., Qin B., Del Giovane C., Li Q., Bauer M., Liu Y., Fang Y., Da Silva T., Zhang Y. (2015). Comparative Efficacy, Acceptability, and Tolerability of Augmentation Agents in Treatment-Resistant Depression. J. Clin. Psychiatry.

[73] Danovich L., Weinreb O., Youdim M.B.H., Silver H. (2011). The involvement of GABAA receptor in the molecular mechanisms of combined selective serotonin reuptake inhibitor-antipsychotic treatment. Int. J. Neuropsychopharmacol..

[74] Cheon E.-J., Lee K.-H., Park Y.-W., Lee J., Koo B.-H., Lee S.-J. (2017). Comparison of the Efficacy and Safety of Aripiprazole Versus Bupropion Augmentation in Patients With Major Depressive Disorder Unresponsive to Selective Serotonin Reuptake Inhibitors: A Randomized, Prospective, Open-Label Study. J. Clin. Psychopharmacol..

[75] Andrade C. (2016). Some augmentation strategies improve outcome but increase discontinuation in adults with treatment-resistant depression. Evid. Based Ment. Health.

[76] Sepede G., De Berardis D., Gambi F., Campanella D., La Rovere R., D’Amico M., Ferro F.M. (2006). Olanzapine Augmentation in Treatment-Resistant Panic Disorder: A 12-Week, Fixed-Dose, Open-Label Trial. J. Clin. Psychopharmacol..

[77] Correll C.U., Schooler N.R. (2020). Negative Symptoms in Schizophrenia: A Review and Clinical Guide for Recognition, Assessment, and Treatment. Neuropsychiatr. Dis. Treat..

[78] Ali S.N., Bazzano L.A. (2018). Hyponatremia in Association With Second-Generation Antipsychotics: A Systematic Review of Case Reports. Ochsner J..

[79] Leth-Møller K.B., Hansen A.H., Torstensson M., Andersen S.E., Ødum L., Gislasson G., Torp-Pedersen C., Holm E.A. (2016). Antidepressants and the risk of hyponatremia: A Danish register-based population study. BMJ Open.

[80] Prior T.I., Baker G.B. (2003). Interactions between the cytochrome P450 system and the second-generation antipsychotics. J. Psychiatry Neurosci..

[81] Bertilsson L., Carrillo J.A., Dahl M.L., Llerena A., Alm C., Bondesson U., Lindstrom L., De La Rubia I.R., Ramos S., Benitez J. (1994). Clozapine disposition covaries with CYP1A2 activity determined by a caffeine test. Br. J. Clin. Pharmacol..

[82] Heeringa M., Beurskens R., Schouten W., Verduijn M.M. (1999). Elevated plasma levels of clozapine after concomitant use of fluvoxamine. Pharm. World Sci..

[83] Chong S.A., Tan C.H., Lee H.S. (1997). Worsening of psychosis with clozapine and selective serotonin reuptake inhibitor combination: Two case reports. J. Clin. Psychopharmacol..

[84] Kennedy W.K., Jann M.W., Kutscher E.C. (2013). Clinically Significant Drug Interactions with Atypical Antipsychotics. CNS Drugs.

[85] Haring C., Meise U., Humpel C., Saria A., Fleischhacker W.W., Hinterhuber H. (1989). Dose-related plasma levels of clozapine: Influence of smoking behaviour, sex and age. Psychopharmacology.

[86] Wetzel H., Anghelescu I., Szegedi A., Wiesner J., Weigmann H., Hartter S., Hiemke C. (1998). Pharmacokinetic interactions of clozapine with selective serotonin reuptake inhibitors: Differential effects of fluvoxamine and paroxetine in a prospective study. J. Clin. Psychopharmacol..

[87] Carrillo J.A., Christensen M., Ramos S.I., Alm C., Dahl M.-L., Benítez J., Bertilsson L. (2000). Evaluation of Caffeine as an In Vivo Probe for CYP1A2 Using Measurements in Plasma, Saliva, and Urine. Ther. Drug Monit..

[88] Rostami-Hodjegan A., Amin A.M., Spencer E.P., Lennard M.S., Tucker G.T., Flanagan R.J. (2004). Influence of dose, cigarette smoking, age, sex, and metabolic activity on plasma clozapine concentrations: A predictive model and nomograms to aid clozapine dose adjustment and to assess compliance in individual patients. J. Clin. Psychopharmacol..

[89] Frick A., Kopitz J., Bergemann N. (2003). Omeprazole reduces clozapine plasma concentrations. A case report. Pharmacopsychiatry.

[90] Khan A.Y., Preskorn S.H. (2005). Examining concentration-dependent toxicity of clozapine: Role of therapeutic drug monitoring. J. Psychiatr. Pract..

[91] Cohen L.G., Chesley S., Eugenio L., Flood J.G., Fisch J., Goff D.C. (1996). Erythromycin-induced clozapine toxic reaction. Arch. Intern. Med..

[92] Hägg S., Spigset O., Mjörndal T., Granberg K., Persbo-Lundqvist G., Dahlqvist R. (1999). Absence of interaction between erythromycin and a single dose of clozapine. Eur. J. Clin. Pharmacol..

[93] Raaska K., Neuvonen P.J. (1998). Serum concentrations of clozapine and N-desmethylclozapine are unaffected by the potent CYP3A4 inhibitor itraconazole. Eur. J. Clin. Pharmacol..

[94] von Moltke L.L., Greenblatt D.J., Granda B.W., Grassi J.M., Schmider J., Harmatz J.S., von Moltke L.L. (1999). Nefazodone, meta-chlorophenylpiperazine, and their metabolites in vitro: Cytochromes mediating transformation, and P450-3A4 inhibitory actions. Psychopharmacology.

[95] Taylor D., Bodani M., Hubbeling A., Murray R. (1999). The effect of nefazodone on clozapine plasma concentrations. Int. Clin. Psychopharmacol..

[96] Spina E., de Leon J. (2007). Metabolic Drug Interactions with Newer Antipsychotics: A Comparative Review. Basic Clin. Pharmacol. Toxicol..

[97] Ring B.J., Binkley S.N., Vandenbranden M., Wrighton S.A. (1996). In vitro interaction of the antipsychotic agent olanzapine with human cytochromes P450 CYP2C9, CYP2C19, CYP2D6 and CYP3A. Br. J. Clin. Pharmacol..

[98] Shin J.G., Soukhova N., Flockhart D.A. (1999). Effect of antipsychotic drugs on human liver cytochrome P-450 (CYP) isoforms in vitro: Preferential inhibition of CYP2D6. Drug Metab. Dispos..

[99] Spina E., de Leon J. (2014). Clinically relevant interactions between newer antidepressants and second-generation antipsychotics. Expert Opin. Drug Metab. Toxicol..

[100] Centorrino F., Baldessarini R.J., Frankenburg F.R., Kando J., Volpicelli S.A., Flood J.G. (1996). Serum levels of clozapine and norclozapine in patients treated with selective serotonin reuptake inhibitors. Am. J. Psychiatry.

[101] Hefner G., Shams M.E.E., Unterecker S., Falter T., Hiemke C. (2016). Inflammation and psychotropic drugs: The relationship between C-reactive protein and antipsychotic drug levels. Psychopharmacology.

[102] Stanke-Labesque F., Gautier-Veyret E., Chhun S., Guilhaumou R. (2020). Inflammation is a major regulator of drug metabolizing enzymes and transporters: Consequences for the personalization of drug treatment. Pharmacol. Ther..

[103] Tio N., Schulte P.F.J., Martens H.J.M. (2021). Clozapine Intoxication in COVID-19. Am. J. Psychiatry.

[104] Thompson D., Delorme C.M., White R.F., Honer W.G. (2021). Elevated clozapine levels and toxic effects after SARS-CoV-2 vaccination. J. Psychiatry Neurosci. JPN.

[105] Ben Dhia A., Hamzaoui S., Mouaffak F. (2020). Epidémie au nouveau coronavirus (SARS-CoV-2) et prescription de la clozapine: Quelles mesures? Pourquoi?. L’Encephale.

[106] Brøsen K., Skjelbo E., Rasmussen B.B., Poulsen H.E., Loft S. (1993). Fluvoxamine is a potent inhibitor of cytochrome P4501A2. Biochem. Pharmacol..

[107] Szegedi A., Anghelescu I., Wiesner J., Schlegel S., Weigmann H., Härtter S., Hiemke C., Wetzel H. (1999). Addition of low-dose fluvoxamine to low-dose clozapine monotherapy in schizophrenia: Drug monitoring and tolerability data from a prospective clinical trial. Pharmacopsychiatry.

[108] Baumann P., Rochat B. (1995). Comparative pharmacokinetics of selective serotonin reuptake inhibitors: A look behind the mirror. Int. Clin. Psychopharmacol..

[109] Nemeroff C.B., Devane C.L., Pollock B.G. (1996). Newer antidepressants and the cytochrome P450 system. Am. J. Psychiatry.

[110] Dequardo J.R., Roberts M. (1996). Elevated clozapine levels after fluvoxamine initiation. Am. J. Psychiatry.

[111] Dumortier G., Lochu A., De Melo P.C., Ghribi O., Roche-Rabreau D., Degrassat K., Desce J.M. (1996). Elevated clozapine plasma concentrations after fluvoxamine initiation. Am. J. Psychiatry.

[112] Lu M.L., Lane H.Y., Lin S.K., Chen K.P., Chang W.H. (2004). Adjunctive fluvoxamine inhibits clozapine-related weight gain and metabolic disturbances. J. Clin. Psychiatry.

[113] Haring C., Neudorfer C., Schwitzer J., Hummer M., Saria A., Hinterhuber H., Fleischhacker W.W. (1994). EEG alterations in patients treated with clozapine in relation to plasma levels. Psychopharmacology.

[114] Freudenreich O., Weiner R.D., McEvoy J.P. (1997). Clozapine-induced electroencephalogram changes as a function of clozapine serum levels. Biol. Psychiatry.

[115] Spina E., Avenoso A., Salemi M., Facciola G., Scordo M.G., Ancione M., Madia A. (2000). Plasma concentrations of clozapine and its major metabolites during combined treatment with paroxetine or sertraline. Pharmacopsychiatry.

[116] Avenoso A., Facciolà G., Campo G.M., Fazio A., Spina E. (1998). Determination of clozapine, desmethylclozapine and clozapine N-oxide in human plasma by reversed-phase high-performance liquid chromatography with ultraviolet detection. J. Chromatogr. B Biomed. Appl..

[117] Jeppesen U., Gram L.F., Vistisen K., Loft S., Poulsen H.E., Brøsen K. (1996). Dose dependent inhibition of CYP1A2, CYP2C19 and CYP2D6 by citalopram, fluoxetine, fluvoxamine and paroxetine. Eur. J. Clin. Pharmacol..

[118] Sindrup S.H., Brøsen K., Gram L.F. (1992). Pharmacokinetics of the selective serotonin reuptake inhibitor paroxetine: Nonlinearity and relation to the sparteine oxidation polymorphism. Clin. Pharmacol. Ther..

[119] Bablenis E., Weber S.S., Wagner R.L. (1989). Clozapine: A novel antipsychotic agent. DICP Annals Pharmacother..

[120] DeVane C.L. (1994). Pharmacokinetics of the newer antidepressants: Clinical relevance. Am. J. Med..

[121] Pollock B.G. (1994). Recent developments in drug metabolism of relevance to psychiatrists. Harv. Rev. Psychiatry.

[122] Popli A., Baldessarini R.J., Cole J.O. (1995). Interactions of Serotonin Reuptake Inhibitors with Tricyclic Antidepressants-Reply. Arch. General Psychiatry.

[123] Slaughter R.L., Edwards D.J. (1995). Recent Advances: The Cytochrome P450 Enzymes. Ann. Pharmacother..

[124] Gorski J., Jones D.R., Wrighton S.A., Hall S.D. (1994). Characterization of dextromethorphan N-demethylation by human liver microsomes. Biochem. Pharmacol..

[125] Jerling M., Lindstrm L., Bondesson U., Bertilsson L. (1994). Fluvoxamine inhibition and carbamazepine induction of the metabolism of clozapine: Evidence from a therapeutic drug monitoring service. Ther. Drug Monit..

[126] Spina E., Avenoso A., Facciolà G., Fabrazzo M., Monteleone P., Maj M., Caputi A.P. (1998). Effect of fluoxetine on the plasma concentrations of clozapine and its major metabolites in patients with schizophrenia. Int. Clin. Psychopharmacol..

[127] Ferslew K.E., Hagardorn A.N., Harlan G.C., McCormick W.F. (1998). A fatal drug interaction between clozapine and fluoxetine. J. Forensic Sci..

[128] Rahman M.S., Grace J.J., Pato M.T., Priest B. (1998). Sertraline in the treatment of clozapine-induced obsessive-compulsive behavior. Am. J. Psychiatry.

[129] Baker R.W., Chengappa K.N.R., Baird J.W., Steingard S., Christ M.A.G., Schooler N.R. (1992). Emergence of obsessive compulsive symptoms during treatment with clozapine. J. Clin. Psychiatry.

[130] Taylor D., Ellison Z., Ementon Shaw L., Wickham H., Murray R. (1998). Co-administration of citalopram and clozapine: Effect on plasma clozapine levels. Int. Clin. Psychopharmacol..

